# Correlation between the severity of COVID-19 vaccine-related adverse events and the blood group of the vaccinees in Saudi Arabia: A web-based survey

**DOI:** 10.3389/fphar.2022.1006333

**Published:** 2022-11-17

**Authors:** Ohoud S. Almalki, Amany S. Khalifa, Ozouf F. Alhemeidi, Ashraf A. Ewis, Abeer M. Shady, Sayed F. Abdelwahab

**Affiliations:** ^1^ Department of Clinical Pharmacy, College of Pharmacy, Taif University, Taif, Saudi Arabia; ^2^ Department of Pharmaceutics and Industrial pharmacy, College of Pharmacy, Taif University, Taif, Saudi Arabia; ^3^ Department of Public Health, Faculty of Health Sciences-AlQunfudah, Umm Al-Qura University, Makkah, Saudi Arabia; ^4^ Department of Public Health and Occupational Medicine, Faculty of Medicine, Minia University, El-Minia, Egypt

**Keywords:** COVID-19, vaccines, severity, blood group, community, Saudi Arabia, adverse event

## Abstract

**Background:** Recent epidemiological studies have reported an association between the ABO blood group and the acquisition, symptom severity, and mortality rate of coronavirus disease 2019 (COVID-19). However, the association between the ABO blood group antigens and the type and severity of COVID-19 vaccine-related adverse reactions has not been elucidated.

**Patients and Methods:** We conducted a cross-sectional, questionnaire-based study in Saudi Arabia from February to April 2022. The study cohort included adults who had received or were willing to receive at least two doses of a COVID-19 vaccine of any type. We used Chi-square test to assess the association between the ABO blood groups and vaccine-related adverse reactions. *p* values of <0.05 were considered significant.

**Results:** Of the 1180 participants, approximately half were aged 18–30 years old, 69.2% were female, and 41.6% reported their blood group as O. The most frequent COVID-19 vaccine-related adverse reactions were fatigue (65%), pain at the injection site (56%), and headache (45.9%). These adverse reactions demonstrated significant correlations with the education level (*p* = 0.003) and nationality (*p* = 0.018) of the participants following the first dose, with gender (*p* < 0.001) following the second dose, and with the general health status (*p* < 0.001) after all the doses. Remarkably, no correlation was observed between the severity of the vaccine-related adverse reactions and ABO blood groups.

**Conclusion:** Our findings do not support a correlation between the severity of COVID-19 vaccine-related adverse reactions and the ABO blood groups of the vaccinees. The creation of a national database is necessary to account for population differences.

## 1 Introduction

Severe acute respiratory syndrome coronavirus-2 (SARS-CoV-2) has caused a challenging and threatening global disease pandemic known as the coronavirus disease 2019 (COVID-19). It is a highly contagious disease that has disrupted the world’s health and economy. In parallel with the restrictions imposed to prevent viral spread and the trials of repurposed antiviral treatments, there is an accelerated development of vaccines to prevent or restrict potential viral damage.1,2 Thus, the Food and Drug Administration and regulatory bodies in the United States and various countries have granted emergency use authorization to some of these rapidly developed COVID-19 vaccines, with more than 200 ongoing clinical trials for COVID-19 vaccines globally (Moderna COVID -19 Vaccine, 2019; Sunny et al., 2020).

The most prevalent COVID-19 vaccines available are those based on mRNA platforms. Although these vaccines appear to be highly effective, they are also reactogenic, which means that they are likely to cause a noticeable immune response (Liu et al., 2021). The World Health Organization (WHO) defines adverse reactions as “a response to a drug that is noxious and unintended, and which occurs at doses normally used in man for prophylaxis, diagnosis, or therapy of disease, or for the modification of physiological function” (The Importance on Pharmacovigilance, 2002). Mild-to-moderate pain at the injection site was the most prevalent reaction among all 11 COVID-19 vaccine trials, with up to 88% of participants experiencing pain that typically resolved within 24–48 h after onset, with a higher incidence recorded in the younger population than in the older population (Li et al., 2020). Other serious adverse events include thrombotic thrombocytopenia syndrome (TTS) (Islam et al., 2021).

Researchers have identified antibodies that bind to platelet factor 4, similar to those associated with heparin-induced thrombocytopenia, in the absence of any previous heparin exposure.9 Vaccines that are more likely to cause TTS, such as Vaxzevria, should be avoided in younger adults for whom an alternative vaccine is available.8 Moreover, researchers specifically reported delayed intense local reactions in Moderna’s phase III trial in 0.8% and 0.2% of the participants after the first and second doses, respectively. However, there was no mention of whether those who had reactions after the first dose experienced a recurrence after the second dose (Lindsey et al., 2021).

Several host and viral factors play crucial roles in their relationship. Interestingly, a recent epidemiological study reported an association between the ABO blood group type with SARS-CoV-2 acquisition, symptom severity, and related mortality.5 This is thought to be a result of natural antibodies against blood group antigens that may act *via* innate immune mechanisms to neutralize viral particles. 5 Alternatively, blood group antigens could serve as additional receptors for the virus, whereas individuals who are expressing these antigens on epithelial cells would have a high propensity for SARS‐CoV‐2 infection (Li et al., 2020). One study found a higher probability of testing positive for COVID-19 in patients with blood group A (Li et al., 2020). By contrast, researchers observed a lower probability of the infection in patients with blood group O than in the general population (Li et al., 2020). On the contrary, other studies have failed to establish such a correlation (Sunny et al., 2020). Of note, the frequency of ABO blood groups in Saudi Arabia (SA) is as follows O > A > B > AB with Rh Positive predominance.11 Interestingly, Alessa et al., did not find an association between COVID-19 vaccine-related adverse events and ABO blood groups among general surgeons in SA between July 2021 and May 2022. However, that study was limited by the small sample size and lacked generalizability, as the study included only general surgeons who received mRNA-based COVID-19 vaccine (Alessa et al., 2022).

To the best of our knowledge, the association between ABO blood group antigens and the type and severity of COVID-19 vaccine-related adverse events in the Saudi general population has never been investigated and this is also globally true. Thus, this study aimed to investigate the relationship between COVID-9 vaccine-related adverse events of any type and the ABO blood groups in the general population to enable better understanding, prediction, and further management of the disease.

## 2 Materials and methods

### 2.1 Study design and subjects

This cross-sectional, online-questionnaire-based study investigated the correlation between blood group antigens and the type and severity of COVID-19 vaccine-related adverse events in SA. The study included adults aged ≥18 years old and who are willing to receive at least two doses of the COVID-19 vaccine and those who had received at least one dose of a COVID-19 vaccine of any type. Participants aged <18 years and those not welling to receive COVID-19 vaccine were excluded. We distributed a self-administered online questionnaire to a random sample of adult participants (N = 1180) from various cities in all regions of SA. The maximum sample size required to provide statistical power to our study at a confidence level of 95% and a margin of error of 5% was 385. This was calculated using the following equation: n = z2 × p(1—p)/e2, where n is the sample size, z (1.96) is the z-score associated with a level of confidence (95%), p is the sample proportion (0.5) expressed as a decimal, and e (0.05) is the margin of error expressed as a decimal. We enrolled 1180 participants, which is just over three times the calculated sample size, to overcome any possible bias that may originate from the snowball sampling technique and ensure that the responses from participants represented a diverse population. The Scientific Research Ethical Committee at Taif University approved the study (Approval No. 43-172), and participants provided their consent online before submitting their responses.

### 2.2 Data collection

We collected the data from February 2022 to April 2022. We first distributed the questionnaire to participants through social media platforms using Google Forms. In addition, we contacted the deanship of scientific research of public universities in all regions of SA so that they could email the invitation to the study along with the link to the questionnaire using staff members’ and students’ confirmed emails that are available on their database. We secured the data and limited access to the primary investigators.

### 2.3 Questionnaire development and validation

The study questionnaire consisted of three sections, all of which were developed specifically for this study. The first section (eight items) included the demographic characteristics, such as sex, age, nationality, social status, educational level, working sector, having a family member working in the healthcare sector, and residential region. The second section (seven items) addressed the participant’s blood type, Rhesus (Rh) factor, general health status, vaccination status, history of COVID-19 before and after receiving the vaccine, and the presence of common diseases in SA (for example, hypertension, diabetes, obesity, heart diseases, and asthma). The third section 16 items was about adverse events of the vaccines; type, onset, duration, and severity of the adverse events, which the participants rated on a scale of 1–10 after each dose for the three doses when applicable; and the type of vaccine and booster. For optimal analysis, we categorized the severity score of vaccine-related adverse events as follows: mild, 1–3; moderate, 4–7; or severe, 8–10 (Ganesan et al., 2022). We provided the questionnaires in Arabic for optimal comprehension given that the primary language of the participants in SA is Arabic. A panel of four researchers at the College of Pharmacy at Taif University reviewed the questionnaire for clarity, consistency, and appropriateness for the local context. Also, the questionnaire was validated on 25 participants on a field trial, and their data were not included in the analysis. The revised questionnaire contained 31 items.

### 2.4 Statistical analyses

Data were analyzed using the IBM SPSS Statistics for Windows, Version 25.0 (IBM Corp., Armonk, NY, United States). An analysis of descriptive statistics was conducted to illustrate the sociodemographic and other selected characteristics of the respondents, and the frequency distributions for the numerical and categorical variables were presented. Chi-squared testing was calculated for the cross-tabulation of variables-related to the type of vaccine and the severity of its related adverse events with other sociodemographic and clinical characteristics of the participants. A *p*-value of less than 0.05 was considered significant.

## 3 Results

This study included 1180 adult participants. Nearly half of the participants (n = 613) were between 18 and 30 years old, 69.2% (*n* = 817) of them were females, 47.5% (*n* = 561) had a bachelor’s degree, and 59.7% (*n* = 705) were from the Western region of SA. Other sociodemographic data of the study participants are shown in Table 1.

**TABLE 1:** Participants’ demographic characteristics.

Characteristics	Number (%)
Age (years)	
18–30	613 (51.9)
31–40	276 (23.4)
41–50	205 (17.4)
51–60	67 (5.7)
>60	19 (1.6)
Gender	
Male	363 (30.8)
Female	817 (69.2)
Nationality	
Saudi	1061 (89.9)
Non–Saudi	119 (10.1)
Marital status	
Single	591 (50.1)
Married	544 (46.1)
Divorced	32 (2.7)
Widow	13 (1.1)
Highest level of education	
< high school	28 (2.4)
High school	216 (18.3)
Bachelor’s degree	561 (47.5)
Diploma	49 (4.2)
Postgraduate degree	326 (27.6)
Current employment status	
Private sector	104 (8.8)
Government sector	467 (39.6)
Unemployed	609 (51.6)
Having a family member in the healthcare sector	
Yes	488 (41.4)
No	692 (58.6)
Geographic region	
Central	249 (21.1)
Western	705 (59.7)
Eastern	151 (12.8)
Southern	44 (3.7)
Northern	31 (2.6)

Clinical characteristics of the study participants are presented in Table 2. In this regard, around 41.6% (*n* = 491) reported having type O blood group. The Rh factor was positive in up to 41.3% of the participants (*n* = 487), 63.3% of the participants (*n* = 747) reported receiving three doses of a COVID-19 vaccine, and 38.4% (*n* = 453) and 45.2% (*n* = 533) reported having mild and moderate symptoms, respectively, after receiving COVID-19 vaccines. About three-quarters of the participants (*n* = 863) reported receiving the Pfizer vaccine as their first COVID-19 vaccine dose (Table 2).

**TABLE 2:** Participants’ clinical characteristics.

Characteristics	Number (%)
Blood group	
A	276 (23.4)
B	161 (13.6)
AB	49 (4.2)
O	491 (41.6)
Unknown	203 (17.2)
Rh Factor	
Positive	487 (41.3)
Negative	94 (8.0)
Unknown	599 (50.8)
COVID–19 infection status since the start of the pandemic	
Yes	16 (1.4)
No	1164 (98.6)
Vaccination status	
Did not receive any doses	3 (0.3)
Received only one dose	10 (0.8)
Received two doses	416 (35.3)
Received three doses	747 (63.3)
Exempted population	4 (0.3)
General health status	
Very good	656 (55.6)
Good	407 (34.5)
Fair	88 (7.5)
Poor	25 (2.1)
Very poor	4 (0.3)
History of chronic diseases	
Yes	205 (17.4)
No	975 (82.6)
COVID–19 infection post–vaccination	
Yes, but currently healthy	301 (25.5)
Yes, and currently infected	22 (1.8)
No	857 (72.6)
Developing COVID 19 vaccine–related adverse events	
None	46 (3.9)
Mild symptoms	453 (38.4)
Moderate symptoms	533 (45.2)
Severe symptoms	148 (12.5)
Type of COVID–19 vaccine for the first dose	
Pfizer	863 (73.1)
Astra–Zeneca	265 (22.5)
Moderna	17 (1.4)
Johnson and Johnson	1 (0.1)
Don’t know	34 (2.9)

More vaccine-related adverse events were reported with Pfizer vaccine compared with others (73.1% after the first dose, 70% after the second dose, and 50.4% after the third dose, *p* = 0.001). Participants reported moderate symptoms after receiving the second dose of Pfizer vaccine (47.7%, *p* = 0.001). Most of the reported adverse events started within 8 h of receiving the dose and lasted for 1–3 days with all doses of the three types of vaccine that were used in SA (Table 3).

**TABLE 3:** Association of COVID–19 vaccine type and the adverse events for the three doses

Item/Vaccine			Pfizer	Astra–Zeneca	Moderna	*p* –value
1st Dose	Vaccine doses^a^	First dose	863 (73.1)	265 (22.5)	17 (1.4)	0.001
		Second dose	826 (70.0)	219 (18.6)	52 (4.4)	
		Third dose	595 (81.5)	21 (1.8)	114 (9.7)	
	Severity of adverse events	Mild	332 (40.3)	102 (39.1)	6 (42.9)	0.197
		Moderate	393 (47.7)	118 (45.2)	4 (28.6)	
		Severe	99 (12.0)	41 (15.7)	4 (28.6)	
	Start of adverse events	0–8 hours	456 (52.8)	153 (57.7)	8 (47.1)	0.604
		9–16 hours	224 (26.0)	59 (22.3)	4 (23.5)	
		17–24 hours	54 (6.3)	20 (7.5)	1 (5.9)	
		No adverse events	129 (14.9)	33 (12.5)	4 (23.5)	
	Duration of adverse events	<1 day	156 (18.1)	43 (16.2)	3 (17.6)	0.893
		1–3 days	473 (54.8)	154 (58.1)	9 (52.9)	
		4–7 days	55 (6.4)	19 (7.2)	1 (5.9)	
		>7 days	54 (6.3)	15 (5.7)	0 (0.0)	
		No adverse events	52 (6.0)	34 (12.8)	4 (23.5)	
2nd Dose	Severity of adverse events	Mild	304 (40.1)	98 (48.3)	11 (21.6)	0.001
		Moderate	343 (45.3)	73 (36.0)	22 (43.1)	
		Severe	111 (14.6)	32 (15.8)	18 (35.3)	
	Start of adverse events	0–8 hours	364 (45.3)	86 (44.9)	29 (55.8)	0.594
		9–16 hours	215 (26.7)	51 (23.8)	12 (23.1)	
		17–24 hours	52 (6.5)	12 (5.6)	3 (5.8)	
		No adverse events	173 (21.5)	55 (25.7)	8 (15.4)	
	Duration of adverse events	<1 day	163 (19.7)	50 (22.8)	10 (19.2)	0.212
		1–3 days	376 (45.5)	82 (37.4)	25 (48.1)	
		4–7 days	46 (5.6)	15 (6.8)	6 (5.9)	
		>7 days	51 (6.2)	12 (5.5)	4 (7.7)	
		No adverse events	190 (23.0)	60 (27.4)	7 (13.5)	
3rd Dose	Severity of adverse events	Mild	224 (41.6)	5 (27.8)	49 (45.0)	0.033
		Moderate	227 (42.2)	6 (33.3)	35 (32.1)	
		Severe	87 (16.2)	7 (38.9)	25 (22.9)	
	Start of adverse events	0–8 hours	272 (47.6)	8 (44.4)	50 (44.2)	0.861
		9–16 hours	157 (27.4)	4 (22.2)	30 (26.5)	
		17–24 hours	33 (5.8)	1 (5.6)	10 (8.8)	
		No adverse events	110 (19.2)	5 (27.8)	23 (20.4)	
	Duration of adverse events	<1 day	113 (19.7)	2 (9.5)	25 (21.9)	0.182
		1–3 days	271 (45.5)	9 (42.9)	47 (41.2)	
		4–7 days	39 (5.6)	1 (4.8)	13 (11.4)	
		>7 days	36 (6.2)	4 (19.0)	6 (5.3)	
		No adverse events	111 (23.0)	4 (19.0)	22 (19.3)	

^a^ Remaining percentage to complete 100% are those who did not report the vaccine type.

The self-reported adverse events associated with different vaccines at different doses and their frequencies are presented in Table 4. As shown, the most frequent COVID-19 vaccine-related adverse events after the first dose were fatigue (65%), pain at the injection site (56%), and headache (45.9%). More serious adverse events after the first vaccine dose were less common, namely, difficulty of breathing (9.55%), seizures (0.51%), and blood clots (0.77%).

**TABLE 4:** Frequency of adverse events after administration of different vaccine doses.

Vaccine related adverse events	Frequency after 1st dose	Frequency after 2nd dose	Frequency after 3rd dose	Average
	*n* = 1173 (%)	*n* = 1163 (%)	*n* = 747 (%)	*n* = 1173 (%)
None	182 (15.52)	245 (21.07)	146 (19.54)	573 (48.85)
Abdominal pain	41 (3.50)	17 (1.46)	8 (1.07)	66 (5.63)
Heart beats disturbance	53 (4.52)	14 (1.20)	12 (1.61)	79 (6.73)
Chest pain	66 (5.63)	22 (1.89)	9 (1.20)	97 (8.27)
Pain at injection site	657 (56.01)	4 (0.34)	57 (7.63)	718 (61.21)
Fever	514 (43.82)	231 (19.86)	59 (7.90)	804 (68.54)
Headache	537 (45.87)	103 (8.86)	67 (8.97)	707 (60.27)
Diarrhea	38 (3.24)	16 (1.38)	8 (1.07)	62 (5.92)
Sweating	55 (4.69)	21 (1.81)	15 (2.01)	91 (7.76)
Shivering	126 (10.74)	28 (2.41)	11 (1.47)	165 (14.07)
Joint pain	320 (27.28)	163 (14.02)	40 (5.35)	523 (44.59)
Menstrual abnormalities	133 (11.34)	96 (8.25)	19 (2.54)	248 (21.14)
Low blood pressure	23 (1.96)	9 (0.77)	2 (0.27)	34 (2.90)
Blood clots	9 (0.77)	3 (0.26)	1 (0.13)	13 (1.11)
Hands/legs swelling	29 (2.47)	15 (1.29)	8 (1.07)	52 (4.43)
Fatigue	764 (65.13)	300 (25.80)	60 (8.03)	1124 (95.82)
Cold extremities	58 (4.94)	18 (1.55)	17 (2.28)	94 (8.01)
Numbness	58 (4.94)	18 (1.55)	16 (2.14)	92 (7.84)
Skin itchiness/rashes	46 (3.92)	10 (0.86)	10 (1.34)	66 (5.63)
Runny nose/congestion	51 (4.35)	18 (1.55)	9 (1.20)	78 (6.65)
Difficulty of breathing	112 (9.55)	83 (7.14)	44 (5.89)	239 (20.38)
Visual complications	42 (3.58)	12 (1.03)	5 (0.67)	59 (5.03)
Nausea/vomiting	111 (9.46)	53 (4.56)	14 (1.87)	178 (15.17)
Loss of consciousness/dizziness	40 (3.41)	25 (2.15)	8 (1.07)	73 (6.22)
Seizures	6 (0.51)	0 (0)	0 (0)	6 (0.51)
Nose/gingival bleeding	6 (0.51)	1 (0.09)	2 (0.27)	9 (0.77)

Table 5 presents the correlation between COVID-19 vaccine-related adverse events and the different demographic characteristics. Approximately 31.8% of the females experienced moderate vaccine-related adverse events after the second dose compared with only 11.3% of the male participants. A significant correlation was found between COVID-19 vaccine-related adverse events after the first dose of the COVID-19 vaccine and education level (*p* = 0.003) and nationality (*p* = 0.018). We found the same correlation with sex after the second (*p* < 0.001) and third (*p* = 0.002) doses of the vaccine. We made a similar observation regarding the vaccine-related adverse events and the participants’ general health status (*p* < 0.001 for the three doses, Table 6). On the other hand, no correlation was observed between the severity of adverse events and the ABO blood group. However, a significant correlation was noticed with Rh factor after the second dose of the vaccine (*p* = 0.044). The multivariate regression analysis revealed a correlation between the severity of adverse events and gender, nationality, education level, and general health status (Table 7).

**TABLE 5:** Correlation between vaccine-related adverse events and the participants’ demographic characteristics.

Demographic characteristics		First dose			p	Second dose			p	Third dose			p
		Mild	Moderate	Severe		Mild	Moderate	Severe		Mild	Moderate	Severe	
Age	18-30	228 (20.1)	294 (25.9)	73 (6.4)	0.127	220 (20.2)	254 (23.3)	86 (7.9)	0.084	211 (25.1)	143 (19.3)	64 (7.6)	0.081
	31-40	113 (9.9)	115 (10.1)	33 (2.9)		110 (10.1)	107 (9.8)	37 (3.4)		86 (10.2)	76 (9.0)	35 (4.1)	
	41-50	70 (6.1)	99 (8.7)	30 (2.6)		78 (7.1)	85 (7.8)	32 (2.9)		72 (8.5)	63 (7.5)	24 (2.8)	
	51-60	33 (2.9)	20 (1.7)	10 (0.8)		32 (2.9)	19 (1.7)	11 (1.0)		32 (3.8)	14 (1.6)	4 (0.4)	
	>60	9 (0.79)	5 (0.44)	2 (0.17)		12 (1.1)	4 (0.3)	0 (0.0)		12 (1.4)	4 (0.4)	0 (0.0)	
	Total	453 (39.9)	533 (47.0)	148 **(**13.1)		452 (41.6)	469 (43.1)	166 **(**15.3)		413 (49.2)	300 (35.7)	127 **(**15.1)	
Gender	Male	152 (13.4)	150 (13.2)	41 (3.6)	0.141	162 (14.9)	123 (11.3)	38 (3.4)	<0.001	158 (18.8)	88 (10.4)	29 (3.4)	0.002
	Female	301 (26.5)	383 (33.7)	107 (9.4)		290 (26.6)	346 (31.8)	128 (11.7)		255 (26.7)	212 (25.2)	98 (11.6)	
	Total	453 (39.9)	533 (47.0)	148 **(**13.1)		452 (41.6)	469 (43.1)	166 **(**15.3)		413 (49.2)	300 (35.7)	127 **(**15.1)	
Nationality	Saudi	398 (35.0)	492 (43.3)	127 (11.1)	0.018	396 (36.4)	429 (39.4)	152 (13.9)	0.112	360 (42.8)	272 (32.3)	113 (13.4)	0.344
	Non-Saudi	55 (4.8)	41 (3.6)	21 (1.8)		56 (5.1)	40 (3.6)	14 (1.2)		53 (6.3)	28 (3.3)	14 (1.6)	
	Total	453 (39.9)	533 (47.0)	148 **(**13.1)		452 (41.6)	469 (43.1)	166 **(**15.3)		413 (49.2)	300 (35.7)	127 **(**15.1)	
Marital status	Single	223 (19.7)	277 (24.4)	71 (6.3)	0.207	208 (19.1)	246 (22.6)	84 (7.7)	0.069	197 (23.5)	151 (18.0)	60 (7.1)	0.783
	Married	214 (18.9)	238 (21.0)	67 (5.9)		227 (20.9)	206 (19.0)	73 (6.7)		198 (23.6)	136 (16.2)	62 (7.4)	
	Divorced	11 (1.0)	11 (1.0)	9 (0.8)		11 (1.0)	10 (0.9)	9 (0.9)		13 (1.5)	8 (1.0)	5 (0.6)	
	Widowed	5 (0.4)	7 (0.6)	1 (0.1)		6 (0.6)	7 (0.7)	0 (0.0)		5 (0.6)	5 (0.6)	0 (0.0)	
Education level	< high school	15 (1.3)	4 (0.3)	6 (0.5)	0.003	12 (1.1)	10 (0.9)	4 (0.3)	0.671	10 (1.1)	5 (0.5)	2 (2.3)	0.208
	High school	88 (7.7)	104 (9.1)	16 (1.4)		79 (7.2)	85 (7.8)	31 (2.8)		82 (9.7)	47 (5.5)	20 (2.3)	
	Bachelor’s degree	194 (17.1)	276 (24.3)	75 (6.3)		210 (19.3)	239 (21.9)	70 (6.4)		178 (21.1)	155 (18.4)	51 (6.0)	
	Diploma	18 (1.5)	18 (1.5)	7 (0.6)		20 (1.8)	16 (1.4)	7 (0.6)		20 (2.3)	11 (1.3)	5 (0.5)	
	Postgraduate degree	138 (12.1)	131 (11.5)	44 (3.8)		131 (12.0)	119 (10.9)	54 (4.9)		123 (14.6)	82 (9.7)	49 (5.8)	
	Total	453 (39.9)	533 (47.0)	148 (13.1)		452 (41.6)	469 (43.1)	166 (15.3)		413 (49.2)	300 (35.7)	127 (15.1)	
Employment	Private	48 (4.2)	42 (3.7)	13 (1.1)	0.593	44 (4.0)	42 (3.8)	13 (1.1)	0.565	32 (3.8)	30 (3.5)	15 (1.7)	0.659
	Government	181 (15.9)	207 (18.2)	59 (5.2)		184 (16.9)	176 (16.1)	73 (6.7)		178 (21.1)	123 (14.6)	51 (6.0)	
	Unemployed	224 (19.7)	284 (25.0)	76 (6.7)		224 (20.6)	251 (23.0)	80 (7.3)		203 (24.1)	147 (17.5)	61 (7.2)	
	Total	453 (39.9)	533 (47.0)	148 **(**13.1)		452 (41.6)	469 (43.1)	166 **(**15.3)		413 (49.2)	300 (35.7)	127 **(**15.1)	
HCW*	Yes	186 (16.4)	219 (19.2)	62 (5.4)	0.982	189 (17.3)	192 (17.6)	68 (6.2)	0.960	184 (21.9)	132 (15.7)	50 (5.9)	0.578
	No	267 (23.5)	314 (27.6)	86 (7.5)		263 (24.1)	277 (25.4)	98 (9.0)		229 (27.2)	168 (20.0)	77 (9.1)	
	Total	453 (39.9)	533 (47.0)	148 **(**13.1)		452 (41.6)	469 (43.1)	166 **(**15.3)		413 (49.2)	300 (35.7)	127 **(**15.1)	
Region	Central	94 (8.3)	112 (9.9)	36 (3.2)	0.989	107 (9.8)	97 (8.9)	34 (3.1)	0.453	97 (11.5)	50 (6.0)	24 (2.9)	0.445
	Western	275 (24.3)	316 (27.9)	85 (7.5)		257 (23.6)	288 (26.5)	103 (9.5)		239 (28.5)	195 (23.3)	79 (9.4)	
	Eastern	53 (4.7)	71 (6.3)	18 (1.6)		59 (5.4)	49(4.5)	22 (2.0)		50 (6.0)	41 (4.9)	17 (2.0)	
	Northern	14 (1.2)	14 (1.2)	4 (0.4)		12 (1.1)	17 (1.6)	1 (0.1)		11 (1.3)	5 (0.6)	4 (0.5)	
	Southern	17 (1.5)	20 (1.8)	5 (0.5)		17 (1.6)	18 (1.7)	6 (0.6)		16 (1.9)	9 (1.1)	3 (0.4)	
	Total	453 (39.9)	533 (47.0)	148 **(**13.1)		452 (41.6)	469 (43.1)	166 **(**15.3)		413 (49.2)	300 (35.7)	127 **(**15.1)	

Healthcare workers in the family.

**TABLE 6:** Correlation between vaccine–related adverse events and participants’ clinical characteristics.

Clinical character		First dose			p	Second dose			p	Third dose			p
		Mild	Moderate	Severe		Mild	Moderate	Severe		Mild	Moderate	Severe	
Blood Groups	A	103 (9.1)	119 (10.5)	40 (3.5)	0.897	106 (9.8)	107 (9.8)	42 (3.9)	0.997	85 (10.1)	62 (7.4)	34 (4.0)	0.406
	AB	16 (1.4)	27 (2.4)	6 (0.5)		21 (1.9)	18 (1.7)	7 (0.6)		19 (2.3)	16 (1.9)	6 (0.7)	
	B	61 (5.4)	71 (6.3)	22 (1.9)		62 (5.7)	63 (5.8)	23 (2.1)		57 (6.8)	39 (4.6)	19 (2.3)	
	O	194 (17.1)	222 (19.6)	57 (5.0)		186 (17.1)	196 (18.0)	68 (6.3)		174 (20.7)	143 (17.0)	52 (6.2)	
	Unknown	79 (7.0)	94 (8.3)	23 (2.0)		77 (7.1)	85 (7.8)	26 (2.4)		78 (9.3)	40 (4.8)	16 (1.9)	
	Total	453 (39.9)	533 (47.0)	148 (13.1)		452 (41.6)	469 (43.1)	166 (15.3)		413 (49.2)	300 (35.7)	127 (15.1)	
Rhesusfactor
	+ ve	187 (16.5)	217 (19.1)	63 (5.6)	0.716	204 (18.8)	173 (15.9)	67 (6.2)	0.044	168 (20)	134 (16)	54 (6.4)	0.793
	– ve	33 (2.9)	49 (4.3)	9 (0.8)		30 (2.8)	44 (4)	8 (0.7)		32 (3.8)	26 (3.1)	10 (1.2)	
	Unknown	233 (20.5)	267 (23.5)	76 (6.7)		218 (20.1)	252 (23.2)	91 (8.4)		213 (25.4)	140 (16.7)	63 (7.5)	
	Total	453 (39.9)	533 (47.0)	148 (13.1)		452 (41.6)	469 (43.1)	166 (15.3)		413 (49.2)	300 (35.7)	127 (15.1)	
Infection status	Yes	3 (0.3)	11 (1.0)	2 (0.2)	0.295	6 (0.6)	8 (0.7)	1 (0.1)	0.573	8 (1.0)	2 (0.2)	1 (0.1)	0.289
	No	450 (39.7)	522 (46.0)	146 (12.9)		446 (41.0)	461 (42.4)	165 (15.2)		405 (48.2)	298 (35.5)	126 (15)	
	Total	453 (39.9)	533 (47.0)	148 (13.1)		452 (41.6)	469 (43.1)	166 (15.3)		413 (49.2)	300 (35.7)	127 (15.1)	
General health status	Good	151 (13.3)	201 (17.7)	43 (3.8)	<0.001	142 (13.1)	182 (16.7)	55 (5.1)	<0.001	118 (14.0)	119 (14.2)	38 (4.5)	<0.001
	Very good	283 (25)	280 (24.7)	66 (5.8)		286 (26.3)	236 (21.7)	75 (6.9)		267 (31.8)	159 (18.9)	58 (6.9)	
	Poor	1 (0.1)	13 (1.1)	11 (1.0)		3 (0.3)	10 (0.9)	11 (1.0)		5 (0.6)	5 (0.6)	6 (0.7)	
	Very poor	0 (0)	0 (0)	4 (0.4)		0 (0)	0 (0)	4 (0.4)		0 (0)	0 (0)	2 (0.2)	
	Fair	18 (1.6)	39 (3.4)	24 (2.1)		21 (1.9)	41 (3.8)	21 (1.9)		23 (2.7)	17 (2.0)	23 (2.7)	
	Total	453 (39.9)	533 (47.0)	148 (13.1)		452 (41.6)	469 (43.1)	166 (15.3)		413 (49.2)	300 (35.7)	127 (15.1)	
Chronic diseases	Yes	379 (33.4)	440 (38.8)	118 (10.4)	0.710	375 (34.5)	393 (36.2)	127 (11.7)	0.146	338 (40.2)	241 (28.7)	101 (12.0)	0.797
	No	74 (6.5)	93 (8.2)	30 (2.6)		77 (7.1)	76 (7.0)	39 (3.6)		75 (8.9)	59 (7.0)	26 (3.1)	
	Total	453 (39.9)	533 (47.0)	148 (13.1)		452 (41.6)	469 (43.1)	166 (15.3)		413 (49.2)	300 (35.7)	127 (15.1)	

**TABLE 7:** Multivariate regression analysis of adverse events post COVID–19 vaccinations.

First dose		Mild		Moderate		Severe	
		aOR (95% CI)	*p* value	aOR (95% CI)	*p* value	aOR (95% CI)	*p* value
Age group	18–30 y	2.87 (0.98–8.42)	0.054	3.31 (1.14–9.67)	0.028	2.16 (0.67–6.96)	0.198
	> 30 y	1		1		1	
Sex	Female	1.92 (0.94–3.94)	0.074	2.23 (1.09–4.55)	0.029	2.54 (1.14–5.65)	0.023
	Male	1		1		1	
Nationality	Non–Saudi	4.12 (0.89–11.17)	0.071	2.72 (0.58–6.73)	0.205	5.35 (1.09–9.27)	0.039
	Saudi	1		1		1	
Marital status	Unmarried	0.93 (0.39–2.19)	0.863	0.88 (0.37–2.07)	0.765	1.17 (0.46–2.98)	0.743
	Married	1		1		1	
Education	< high school	1.20 (0.26–5.56)	0.816	0.88 (0.19–4.15)	0.871	1.20 (0.21–6.84)	0.837
	High school	0.75 (0.42–1.35)	0.341	0.71 (0.40–1.27)	0.249	0.89 (0.45–1.74)	0.724
	Diploma	0.67 (0.25–1.76)	0.413	0.47 (0.17–1.27)	0.135	0.70 (0.22–2.22)	0.545
	Postgraduate	1.19 (0.68–2.09)	0.541	0.95 (0.54–1.67)	0.859	1.47 (0.79–2.76)	0.226
	Bachelor's degree	1		1		1	
Occupation	Private sector	5.68 (0.69–8.41)	0.105	3.63 (0.44–9.78)	0.231	4.13 (0.47–6.57)	0.203
	Unemployed	0.54 (0.20–1.47)	0.229	0.43 (0.16–1.17)	0.099	0.63 (0.21–1.89)	0.414
	Governmental sector	1		1		1	
Residence	Eastern	0.60 (0.24–1.52)	0.283	0.59 (0.23–1.47)	0.253	0.46 (0.17–1.23)	0.122
	Southern	0.32 (0.10–1.02)	0.054	0.35 (0.11–1.11)	0.074	0.26 (0.07–0.93)	0.093
	Northern	0.62 (0.12–3.12)	0.559	0.96 (0.20–4.74)	0.964	0.16 (0.01–1.96)	0.152
	Central	0.70 (0.12–3.90)	0.681	0.73 (0.13–4.08)	0.719	0.48 (0.07–3.17)	0.442
	Western	1		1		1	
Blood group	B	1.44 (0.53–3.93)	0.472	1.54 (0.57–4.18)	0.395	1.24 (0.41–3.72)	0.699
	AB	1.39 (0.38–5.07)	0.621	1.18 (0.32–4.36)	0.807	1.17 (0.27–4.98)	0.835
	O	1.86 (0.84–4.11)	0.124	1.96 (0.89–4.29)	0.095	1.49 (0.63–3.54)	0.364
	Unknown	1.18 (0.39–3.54)	0.768	1.19 (0.40–3.57)	0.754	0.95 (0.28–3.15)	0.928
	A	1		1		1	
Rh Factor	Negative	1.09 (0.29–4.01)	0.902	1.40 (0.39–5.11)	0.609	0.78 (0.18–3.34)	0.739
	Unknown	1.02 (0.50–2.10)	0.955	0.97 (0.47–1.99)	0.936	0.96 (0.44–2.11)	0.918
	Positive	1		1		1	
COVID–19 infection status since the start of the pandemic	Yes	0.94 (0.56–1.56)	0.799	1.21 (0.73–2.01)	0.457	1.20 (0.68–2.13)	0.524
	No	1		1		1	
	Good	1.17 (0.56–2.45)	0.685	1.62 (0.77–3.39)	0.202	1.43 (0.63–3.22)	0.394
General health status	Fair	0.22 (0.08–0.63)	0.005	0.55 (0.20–1.47)	0.231	1.27 (0.45–3.62)	0.654
	Poor	0.59 (0.33–1.08)	0.086	0.74 (0.38–1.41)	0.357	2.73 (1.44–5.17)	0.002
	Very poor	1.05 (1.05–1.05)	0.137	2.05 (0.78–5.42)	0.147	2.97 (0.41–21.53)	0.282
	Very good	1		1		1	
History of chronic diseases	Yes	1.46 (0.97–2.17)	0.067	1.61 (1.05–2.45)	0.029	1.69 (0.99–2.88)	0.045
	No	1		1		1	

## 4 Discussion

In this study, we investigated the relationship between COVID-19 vaccine-related adverse events and the ABO blood groups. We found no significant correlation between the COVID-19 vaccine-related adverse events and blood groups of the study participants. However, we found a correlation between COVID-19 vaccine-related adverse events and general health status, education level, sex, Rh factor, and nationality of the participants.

The reported adverse events were like those reported in the literature and were non-immunological, which mostly started within 8 h after the vaccination and lasted between 1 and 3 days. Nevertheless, the frequency and type when we looked at the breakdown of adverse events were slightly different between doses. For example, pain or edema at the injection site (56%), headache (45.9%), and fever (43.8%) were among the adverse events our participants most frequently reported after their first dose, which were consistent with other reports.14, 15 However, the frequency of these adverse events after the second and third doses appeared to decrease significantly after each subsequent dose. This is conflicting with what has been reported by the Center for Disease Control and Prevention in the United States, where reactions after the third dose were comparable with those after the second dose (AnneHause et al., 2021). This could be attributable to the participants’ inabilities to recall events after the subsequent COVID-19 vaccine doses or self-medication with analgesic or antipyretic agents, as they had previously received information on mitigation strategies for vaccine-related adverse events, which were well-known to them by the second and third doses. Our results also contradicted what was reported locally, as more adverse events were observed after the second dose of Pfizer/BioNTech vaccine in adults in SA. (Mohammed et al., 2021) (Ahsan et al., 2021) Still, these studies were limited by their small sample size. In addition, we included all types of COVID-19 vaccines in our study and investigated the vaccine-related adverse events including the booster dose of the vaccines, possibly decreasing the total reported adverse events compared with other studies. (Mohammed et al., 2021), (Ahsan et al., 2021).

In our study, younger participants (18–30 years, 52.4% after the first dose) reported adverse events more frequently than older ones (>60 years, 1.4% after the first dose) did, which was consistent with the results of other reports.7,16 However, we had less representation of the older participants in our study.

Considering the correlation with the demographic data after adjustment for other variables, we found a significant relationship with sex after the second and third doses (*p* value were <0.001 and 0.002, respectively). This observation might be driven by the high frequency of severe COVID-19 vaccine-related adverse events (11.7% and 11.6% after the second and the third doses, respectively) that the study participants reported, compared with the first dose (9.4%). Our findings resemble those from local studies, where sex was a predictor of the severity or occurrence of adverse events. Women and younger adults have a more profound vaccine-related responses. Moreover, we found a correlation between nationality and education level after the first dose of the vaccine (*p* = 0.018 and 0.003 for nationality and education level, respectively), which is consistent with the local studies and might be driven by more moderate vaccine-related adverse events among the bachelor’s degree (and above) holders after the first dose.

We did not find any correlation between the severity of adverse events and the ABO blood group among the study participants. This finding is consistent with that of a small local study (*n* = 612) that was conducted among surgeons in SA who received 1–2 doses of mRNA-based COVID-19 vaccines and found no correlation with the blood group (Alessa et al., 2022). Overall, we found a correlation with the general health status, which could be driven by more severe adverse events with all doses. Mohammed et al., found a correlation between reporting adverse events and having known allergies among 397 healthcare providers who participated in that study in SA (Mohammed et al., 2021). Another group of researchers made the same observation in a small study conducted in SA (Ahsan et al., 2021). In neither study did the researchers investigate the correlation between adverse events and general health status. This could be used to identify people at risk for developing adverse events, who would benefit from more frequent monitoring or a preventive self-medication intervention.

One of this study’s strengths is that it is the first investigation of the correlation of COVID-19 vaccine-related adverse events with the ABO blood groups of COVID-19 vaccinees in the community, taking into account the three doses of the COVID-19 vaccine of any type. Second, we included a suitable representation of the study population in SA, as we included adult participants from all regions of SA and various age groups. Third, all the study participants completed all mandatory items of the questionnaire. Nonetheless, this study had a few limitations. The participants may have recall bias and therefore might have inaccurately or incompletely reported their SARS‐CoV‐2 vaccine-related adverse events. Moreover, we noticed a low participation rate among participants from the southern and northern regions of SA and non-Saudi participants, which might affect the generalizability of the results. Fourth, the use of an online survey may not have been objective because the participants might overestimate or underestimate the severity of their self-reported adverse events. Furthermore, nonresponse bias may be present since some participants did not respond to the optional questions related to the severity of the adverse reactions following second and third vaccine doses.

## 5 Conclusion

This study does not support a correlation between COVID-19 vaccine-related adverse events and the ABO blood groups of the vaccinees in SA. The creation of a national database would be necessary to account for population differences. Our results showed that the general pattern of vaccine-related adverse events resembles what has been reported internationally and locally. However, we have not studied the more severe forms, such as anaphylaxis and facial paralysis.

## Data Availability

The raw data supporting the conclusions of this article will be made available by the authors, without undue reservation.

## References

[B1] Ahsan Waquar NabeelSyed K. Alsraeya Aseel A. Alhazmi H. A. Najmi A. Bratty M. A. (2021). Post-vaccination survey for monitoring the side effects associated with COVID-19 vaccines among healthcare professionals of Jazan province, Saudi Arabia. Saudi Med. J. 42 (12), 1341–1352. 10.15537/smj.2021.42.12.20210576 34853140 PMC9149761

[B2] Alessa Mohammed Y. J Aledili Fatimah Alnasser Ahmad A. Aldharman S. S. Al Dehailan A. M. Abuseer H. O. (2022). The side effects of COVID-19 vaccines and its association with ABO blood type Among the general surgeons in Saudi Arabia. Cureus 14 (3), e23628. 10.7759/cureus.23628 35494934 PMC9050174

[B3] AnneHause M. Baggs James Gee Julianne Marquez P. Myers T. R. Shimabukuro T. T. (2021). Safety monitoring of an additional dose of COVID-19 vaccine—United States, august 12–september 19, 2021. MMWR. Morb. Mortal. Wkly. Rep. 70 (39), 1379–1384. 10.15585/mmwr.mm7039e4 34591835 PMC8486391

[B4] Ganesan Subhashini AlKetbi Latifa Nawal AlKaabi Al Mansoori M. Al Maskari N. N. Al Shamsi M. S. (2022). Vaccine side effects following COVID-19 vaccination among the residents of the UAE-an observational study. Front. Public Health 10, 876336. 10.3389/fpubh.2022.876336 35602146 PMC9120526

[B5] Islam Amin Bashir Mohammed S. Joyce Kevin Rashid H. Laher I. Elshazly S. (2021). An update on COVID-19 vaccine induced thrombotic thrombocytopenia syndrome and some management recommendations. Molecules 26 (16), 5004. 10.3390/molecules26165004 34443589 PMC8400504

[B6] Kabrah1 S. M. Flemban A. F. Khogeer A. A. Bawazir W. A. (2021). Reviewing publication discussing the frequency of ABO and rhesus-D blood groups in Saudi Arabia. JMDSR 9 (10), 29–37.

[B7] Li Juyi Wang Xiufang Chen Jian Cai Y. Deng A. Yang M. (2020). Association between ABO blood groups and risk of SARS‐CoV‐2 pneumonia. Br. J. Haematol. 190 (1), 24–27. 10.1111/bjh.16797 32379894 PMC7267665

[B8] Lindsey R. Baden ElSahly Hana M. Essink Brandon Kotloff K. Frey S. Novak R. (2021). Efficacy and safety of the mRNA-1273 SARS-CoV-2 vaccine. N. Engl. J. Med. Overseas. Ed. 384 (5), 403–416. 10.1056/nejmoa2035389 PMC7787219 33378609

[B9] Liu Nanyang Zhang Tingting Ma Lina Zhang H. Wang H. Wei W. (2021). The impact of ABO blood group on COVID-19 infection risk and mortality: A systematic review and meta-analysis. Blood Rev. 48, 100785. 10.1016/j.blre.2020.100785 33309392 PMC7834371

[B10] Meo S. A. Bukhari I. A. Akram J. Meo A. S. Klonoff D. C. (2021). COVID-19 vaccines: Comparison of biological, pharmacological characteristics and adverse effects of pfizer/BioNTech and Moderna vaccines. Eur. Rev. Med. Pharmacol. Sci. 25 (3), 1663–1669. 10.26355/eurrev_202102_24877 33629336

[B11] Moderna COVID-19 Vaccine US Food and drug administration web site. Available at: https://www.fda.gov/emergency-preparedness-and-response/coronavirus-disease-2019-covid-19/moderna-covid-19-vaccine (Accessed October 4, 2022).

[B12] Mohammed Rehab A. Garout Rana M. Wahid Sherehan Ayub F. Firas ZinAlddin L. M. Sultan I. (2021). A survey on the side effects of Pfizer/BioNTech COVID-19 vaccine among vaccinated adults in Saudi Arabia. Cureus 13 (11), e19222. 10.7759/cureus.19222 34873547 PMC8640570

[B13] Nguyen Thi-Huong Medvedev Nikolay Delcea Mihaela Greinacher A. (2017). Anti-platelet factor 4/polyanion antibodies mediate a new mechanism of autoimmunity. Nat. Commun. 88 (1). 10.1038/ncomms14945 PMC5458132 28530237

[B14] Pfizer-BioNTech COVID-19 Vaccine US Food and drug administration web site. Available at: https://www.fda.gov/emergency-preparedness-and-response/coronavirus-disease-2019-covid-19/pfizer-biontech-covid-19-vaccines (Accessed October 4, 2022).

[B15] Sunny Dzik Kent Eliason Morris Edward B. Kaufman R. M. North C. M. (2020). COVID‐19 and ABO blood groups. Transfusion 60 (8), 1883–1884. 10.1111/trf.15946 32562280 PMC7323215

[B16] The Importance on Pharmacovigilance (2002). Safety monitoring on medicinal products. World health organization (WHO) web site. Available at https://apps.who.int/iris/bitstream/handle/10665/42493/a75646.pdf?sequence=1&isAllowed=y (Accessed October 4, 2022).

